# The Workmen's Compensation Act

**Published:** 1898-04-30

**Authors:** 


### THE WORKMEN'S COMPENSATION ACT.

A memorandum has been issued from the Home Office giving details as to the duties and emoluments of the medical referees who will shortly be appointed under the Workmen's Compensation Act, 1897. Referees will be appointed for each of the 55 circuits into which England and Wales are divided. In some cases the circuits will be subdivided, but the County Court judge or the County Court arbitrator is left free to select such of the referees for the circuit as he sees fit. The following scale of fees has been approved of by the Treasury:—

| | |
|---|---|
| For a first reference, to include examination and written report ... ... ... ... | 2 guineas. |
| For a further written statement on the same reference ... ... ... ... ... ... | 1 guinea. |
| For a second or subsequent reference in the same case ... ... ... ... ... ... | 1 guinea. |
| For attendance at the proceedings on the request of a County Court judge or County Court arbitrator at a time and place to be arranged ... ... ... ... ... ... ... | 3 guineas. |

Where the medical referee has to travel to a place distant more than two miles there will be a further fee of 5s. a mile.

